# Folate-deficiency induced acyl-CoA synthetase short-chain family member 2 increases lysine crotonylome involved in neural tube defects

**DOI:** 10.3389/fnmol.2022.1064509

**Published:** 2023-01-20

**Authors:** Shan Wang, Yubing Zeng, Xuejia He, Fan Liu, Pei Pei, Ting Zhang

**Affiliations:** ^1^Beijing Municipal Key Laboratory of Child Development and Nutriomics, Capital Institute of Pediatrics, Beijing, China; ^2^Capital Institute of Pediatrics-Peking University Teaching Hospital, Beijing, China; ^3^Graduate School of Peking Union Medical College, Capital Institute of Pediatrics, Beijing, China

**Keywords:** neural tube defect, crotonylation, folate-deficiency, ACSS2, Kcr

## Abstract

Maternal folate deficiency increases the risk of neural tube defects (NTDs), but the mechanism remains unclear. Here, we established a mouse model of NTDs *via* low folate diets combined with MTX-induced conditions. We found that a significant increase in butyrate acid was observed in mouse NTDs brains. In addition, aberrant key crotonyl-CoA-producing enzymes acyl-CoA synthetase short-chain family member 2 (ACSS2) levels and lysine crotonylation (Kcr) were elevated high in corresponding low folate content maternal serum samples from mouse NTD model. Next, proteomic analysis revealed that folate deficiency led to global proteomic modulation, especially in key crotonyl-CoA-producing enzymes, and dramatic ultrastructural changes in mouse embryonic stem cells (mESCs). Furthermore, we determined that folate deficiency induced ACSS2 and Kcr in mESCs. Surprisingly, folic acid supplementation restored level of ACSS2 and Kcr. We also investigated overall protein post-translational Kcr under folate deficiency, revealing the key regulation of Kcr in glycolysis/gluconeogenesis, and the citric acid cycle. Our findings suggest folate deficiency leads to the occurrence of NTDs by altering ACSS2. Protein crotonylation may be the molecular basis for NTDs remodeling by folate deficiency.

## 1. Introduction

Neural tube defects (NTDs) are serious birth defects of the central nervous system caused by the failure of neural tube closure (NTC) during embryonic development. NTDs are the most common birth defect affecting population quality in China and countries worldwide. NTDs mainly occur in the spinal cord and brain (1991). Data from 2015 showed that NTDs occurred in 18.6 cases per 10,000 fetuses worldwide on average; ~ 50% of families chose to terminate the pregnancy, and 75% of NTD patients survived beyond the age of five (Wilde et al., 2014). NTDs result from the interaction between genetic and environmental factors. NTC may be affected by environmental factors through a direct impact on embryonic metabolic biology. Globally, countries have achieved remarkable results with fortified folic acid foods in preventing NTDs (Imbard et al., 2013). Folic acid levels in pregnant women are highly correlated with NTC. Carbon metabolism has received particular attention, especially because maternal folic acid supplementation was found to reduce the risk of NTDs (MRC Vitamin Study Research Group, 1991). Abnormalities in thymine and purine biosynthesis were found in NTD mouse models and some cases of NTDs. Current data show that folic acid deficiency may be only a modest component of NTD risk, and the underlying mechanisms of NTDs remain unclear (De Marco et al., 2011).

The metabolism of carbohydrates, lipids, and proteins is integrated through common intermediate metabolites, specifically the intermediate products of the convergence of two metabolic pathways: the tricarboxylic acid cycle (TCA) and bio-oxidation and then anabolism and catabolism. Current findings suggest that some intermediate metabolites, such as acetyl-CoA, A-ketoglutarate, and S-adenosylmethionine, serve as substrates or cofactors for enzymes that regulate histone modification (Cai et al., 2011). These include the covalent modification of histones, such as 2-hydroxyisobutyrylation, croton acylation, β-hydroxybutyrylation, and O-GlcNA acylation, which affect gene expression. Similarly, an overall decrease in nuclear acetyl CoA levels reduces histone acetylation, whereas a reduction in NAD + levels inhibit histone deacetylation (Shiraki et al., 2014). Some metabolites derived from short-chain fatty acids, such as propionyl-CoA, crotonyl-CoA, and butyryl-CoA, have been demonstrated to be substrates for histone and some non-histone lysine acylation(Choudhary et al., 2014). Post-translational modifications (PTMs) that depend on lysine acylation are functionally relevant and strengthen the link between metabolism and cellular functions. In addition, lysine crotonylation (Kcr) in histones activates gene expression through some unknown mechanism, as can a number of non-histone protein(Tan et al., 2011). Protein PTMs maintain cell homeostasis by regulating protein stability, enzyme activity, and protein interactions, and abnormal PTMs can lead to pathological conditions. A newly identified set of short-chain lysine acylations (including butyrylation, propionylation, succinylation, glutarylation, crotonylation, hydroxybutyrylation, and malonylation) have a similar structure to the known lysine acetylation, but their functions are not completely clear (Tan et al., 2011). Recently, the PTM of histones by Kcr was found to be involved in the regulation of host gene expression (Sabari et al., 2017; Zhao et al., 2018). Several ‘writers’ of histone crotonylation have also been reported (Sabari et al., 2017), including the crotonyl-CoA-producing enzyme acyl-CoA synthetase short-chain family member 2 (ACSS2). The ACSS2 enzyme is an important component of fatty acid metabolism in the gastrointestinal tract and plays an essential role in lipid homeostasis (Carey et al., 2015). However, whether crotonylation is involved in NTDs, and whether folic acid levels affect the development of NTDs by influencing the relationship between ACSS2 and crotonylation remain unknown.

Some evidences imply that metabolic programs are related to epigenetic states in specific embryo development (Carey et al., 2015). More recently, the epigenetic regulation of gene expression has been identified as a potential pathogenic factor of human NTDs (Frey and Hauser, 2003). Animal experiments show that protein modification or histone modification regulates the expression of molecules necessary for NTC. Several histone modifications have been shown to be involved in NTDs and affect NTD-related gene activation and silencing, such as acetylation, methylation, phosphorylation, and ubiquitination. Epigenetic regulators play a key role in mouse NTC and may be affected by folic acid (Cheng et al., 2019), suggesting that genome-wide analysis may reveal NTD-related histone or DNA methylation epigenome changes. Advanced experimental techniques can be used to evaluate the folic acid deficiency-induced transcriptional of specific cell types and correlate this with changes in histone ubiquitination and phosphorylation and advanced chromatin status (Xie et al., 2017; Pei et al., 2019). Our previous results suggest that high homocysteine levels may increase histone homocysteinylation, resulting in the decreased expression of some NTC-related genes and leading to NTD formation (Zhang et al., 2018). These findings suggest that metabolic control an important epigenetic mark in alters gene expression and play a role in NTC at the time of neurulation.

In this study, we used a quantitative proteomics approach to obtain a global view of the proteome and crotonylome alterations in response to folate deficiency in mouse embryonic stem cells (mESCs). Proteomics analysis showed that folate deficiency caused global proteomic changes in folate-free mESCs, particularly induced key crotonyl-CoA-producing enzymes ACSS2 and Kcr. We also investigated global protein post-translational Kcr under folate deficiency in mESCs, which revealed key regulatory roles of Kcr in glycolysis/gluconeogenesis, and the citrate cycle (TCA cycle). Furthermore, we found low folate levels and higher ACSS2 levels in the corresponding maternal serum samples in mouse NTD model. This finding was validated in folate deficiency-induced mouse NTD models, indicating that abnormal ACSS2 expression and crotonylation may participate in neural development and contribute to the occurrence of NTDs.

## 2. Results

### 2.1. Folate-deficiency induced ACSS2 levels in mice with NTDs

**Figure 1:**
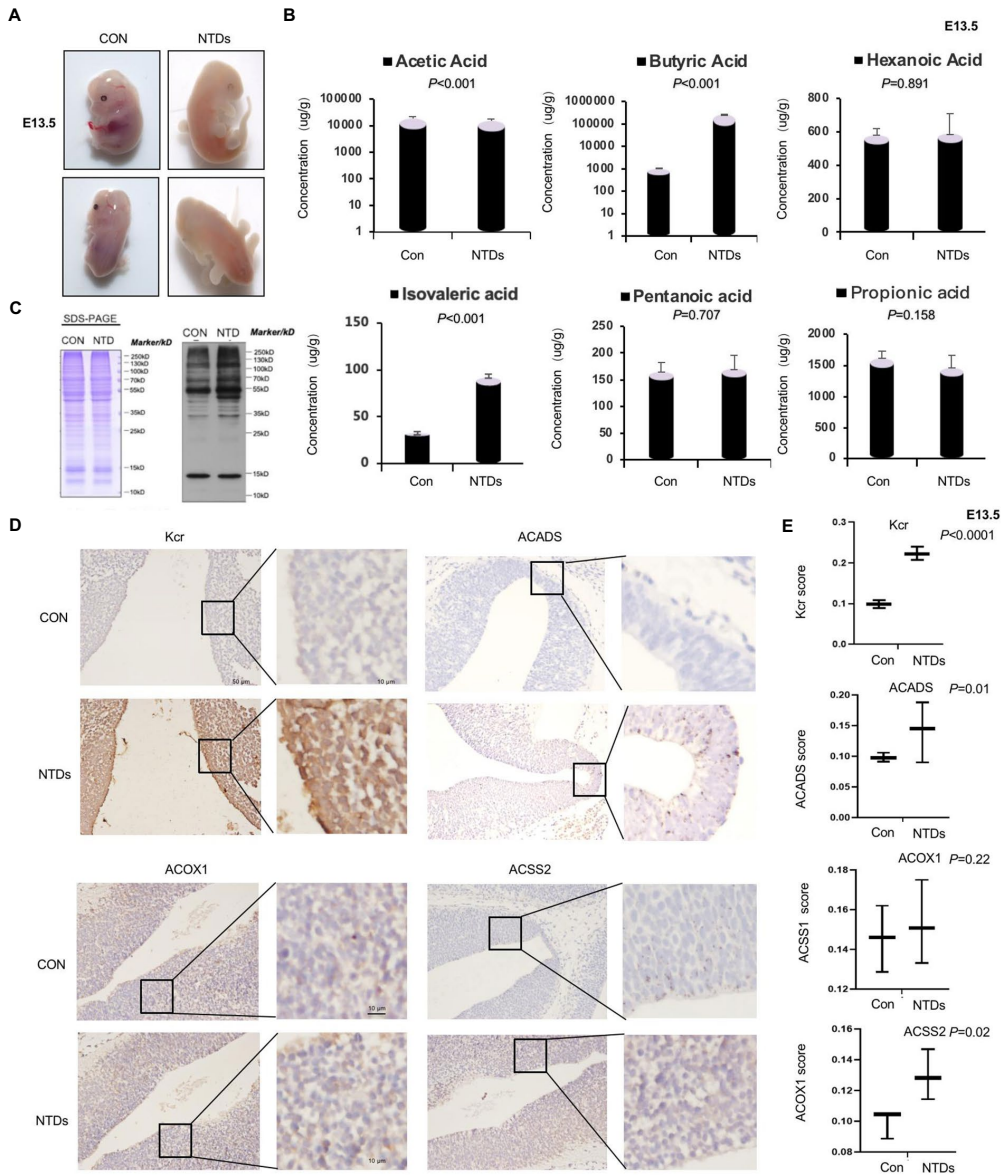
Folate deficiency reduced ACSS2 levels in mouse Neural tube defects (NTDs). **(A)** Methotrexate (MTX) and folate deficiency-induced NTDs in C57BL/6 mouse embryos at 13.5 day. **(B)** SDS-PAGE analysis of lysates of cranial neural tissue in normal and folate deficiency-induced mouse NTDs harvested at E13.5. Western blot analysis of tissue lysate with anti-crotonyllysine antibodies. **(C)** Targeted short-chain fatty acid detection in the cranial neural tissue of normal and folate deficiency-induced mouse NTDs harvested at E13.5 (*n* = 6). Analysis of short-chain fatty acids based on the LC–MS/MS platform and absolute quantitative detection of seven short-chain fatty acids: acetic acid, propionic acid, isobutyric acid, isovaleric acid, pentanoic acid, and hexanoic acid. According to the sampling and/or dilution times and other parameters, the metabolite concentration was further calculated, and finally, the absolute content of each metabolite in the actual samples was obtained. **(D)** Representative images of immunohistochemical staining of Kcr, ACSS2, ACOX1, and ACADS in the cranial neural tissue from E13.5 embryos. **(E)** Kcr, ACSS2, ACOX1, and ACADS expression scores are shown as box plots, with the horizontal lines representing the median. Data are the mean ± SD. (*n* = 3), ^*^p < 0.05, by Student’s *t*-test.

In our previous study, NTDs mouse model was freshly established *via* intraperitoneal injection of MTX (1.5 mg/kg) at E7.5 under low-folate diet conditions (Pei et al., 2019; Wang et al., 2022). Here, we established this NTD mouse model. As shown in the Figure 1A, the embryos of mice in the normal feeding group were plump and smooth in appearance, with well-developed ventricles and complete neural tube closure. Low folic acid feeding combined with MTX induced the abnormality rate phenotype of the mouse model. We found failure of closure at the level of the hindbrain/cervical boundary at this stage leads to craniorachischisis. The survival rate of mouse embryo was 18.2% (10/55), the absorption rate was 9.1% (5/55), and the malformation rate was 36.3% (20/55). Level of folate in maternal serum was significantly decreased in our folate-deficiency mouse model, suggesting that folate deficiency affected neurogenesis in fetuses during early pregnancy (Supplementary Figure S1A). Folate levels were also decreased in tissue of NTDs fetal brain (Supplementary Figure S1B). Next, we quantified short-chain fatty acids, including acetic acid, propionic acid, butyric acid, isovaleric acid, pentanoic acid, and hexanoic acid, using the LC–MS/MS platform (Supplementary Table S1). The results showed that butyric acid was significantly increased by 100-fold, and isovaleric acid was increased by 3-fold, acetic acid was decreased slightly. However, no significant changes in propionic acid, pentanoic acid, or hexanoic acid were detected (Figure 1B). Protein modification in brain tissue of NTDs was confirmed by western blotting with anti-crotonyllysine antibodies. We also found that crotonyllysines were elevated in MTX-induced cranial neural brain tissue from E13.5 embryos (Figure 1C). We next asked whether the level of Kcr, ACSS2 and other two enzymes ACADS and ACOX1 that catalyze conversion of butyryl-CoA to crotonyl-CoA is eccentrically changed in cranial neural tissue. By this, we examined Kcr, ACSS2, ACADS, and ACOX1 levels of cranial neural tissues in triplicate and matched with normal tissues by immunohistochemical analysis. The staining of total Kcr and ACSS2 was increased in mouse NTD samples compared with that in normal tissues (Figures 1D, E). There was no statistically significant difference in ACOX1. Next, six pairs including human fetuses with spina bifida were selected, and the gestational weeks and sex were summarized in Supplementary Figure S1C. Folate levels were lower in fetuses with NTDs than in the controls (Supplementary Figure S1C). Western blot analysis also revealed that ACSS2 was increased in NTDs fetuses significantly (Supplementary Figures S1D, E). Taken together, our findings suggest that the ACSS2 upregulation is relevant to the occurrence of folate deficient-induced mouse NTDs, likely through the regulation of Kcr levels.

### 2.2. Identification of numerous proteins differentially expressed in folate-free mESCs

**Figure 2:**
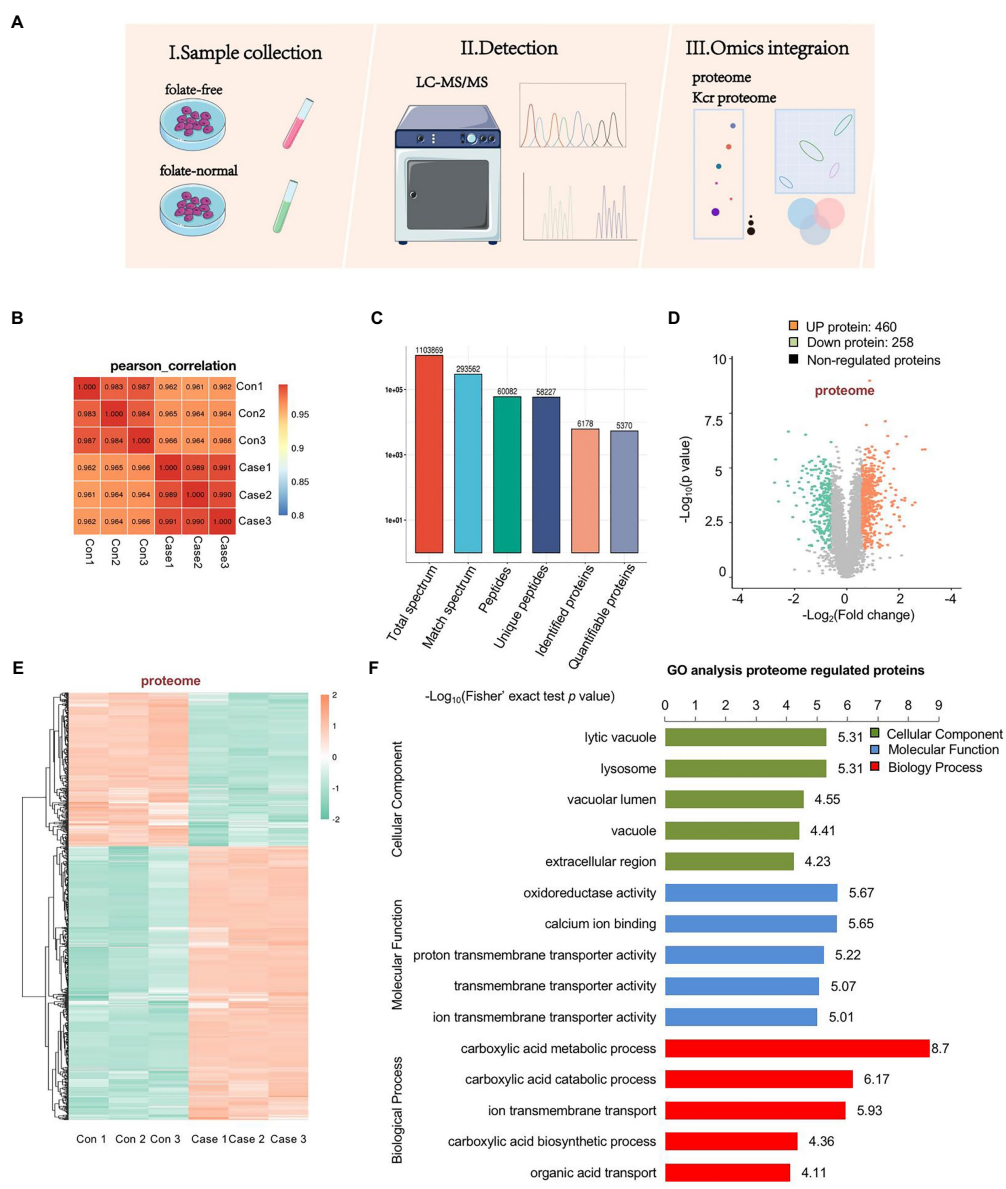
Profiling proteomics of folate-free mESCs. **(A)** The flow chart depicted the experimental procedures and data analysis workflow. **(B)** Heat maps are drawn with Pearson correlation coefficients between all samples. Pearson correlation coefficient is negative correlation when it is close to −1, positive correlation when it is close to 1, and no correlation when it is close to 0. **(C)** An overview of protein identification. **(D)** Volcano plot showing double thresholds for DEPs in normal folate (4 mg/l) versus folate-free conditions (0 mg/l). Green dots indicate a decrease protein, and red indicates an increase protein in abundance (FC ≥ 1.40 or ≤ 0.71, p < 0.05). **(E)** Hierarchical clustering heatmap depicting individual samples and protein expression differences between normal folate versus folate-free mESCs (n = 3 per group). **(F)** Bar chart of the DEPs organized by enriched BP, CC, and MF, as determined with the Database for Annotation, Visualization, and Integrated Discovery (DAVID).

To assess the effect of folate deficiency, we ran protein extractions of mouse ESCs cultured with normal folate (4 mg/l; FA4) and folate-free (0 mg/l; FA0) through LC–MS/MS (Figure 2A). The soluble proteins obtained from both (FA4) and (FA0) mESCs were separately analyzed for identification with three replications. Pearson’s correlation of the Log_2_ LFQ intensity between replications was 1 (Figure 2B). The identified protein contained at least one unique peptide. The distribution of identified peptide length through mass spectrometry fulfilled the demand of quality control (Supplementary Figure S2). In total, 6,178 proteins were identified, of which 5,370 proteins were quantifiable (Figure 2C). To identify differentially expressed proteins (DEPs), the cutoffs of the fold change in abundance and *p* value were set to 1.5 and 0.05, respectively. Among DEPs, 718 proteins (460 up-regulated proteins and 258 down-regulated proteins) were remarkably modulated in folate-free mESCs (Figure 2D; Supplementary Table S2). Heatmap demonstrated that folate deficiency persuaded a broad proteome modulation in mESCs (Figure 2E). To explore host functions by folate deficiency, Gene Ontology (GO) enrichment was performed to identify biological processes (BP), cellular components (CC) and molecular functions (MF) enriched among significantly regulated proteins, as shown in Figure 2F and Supplementary Table S3. According to *p* values achieved the fisher’s exact test, the top three ranked ahead biological process for DEPs were as follows: carboxylic acid metabolic processes, carboxylic acid catabolic processes, and ion transmembrane transport. The top three cellular components were the lysosome, lytic vacuole, and vacuolar lumen. The top three molecular functions associated with these proteins were as follows: oxidoreductase activity, calcium ion binding, and proton transmembrane transporter activity.

### 2.3. Key crotonyl-CoA-producing enzymes are induced and enriched in folate-free mESCs

**Figure 3:**
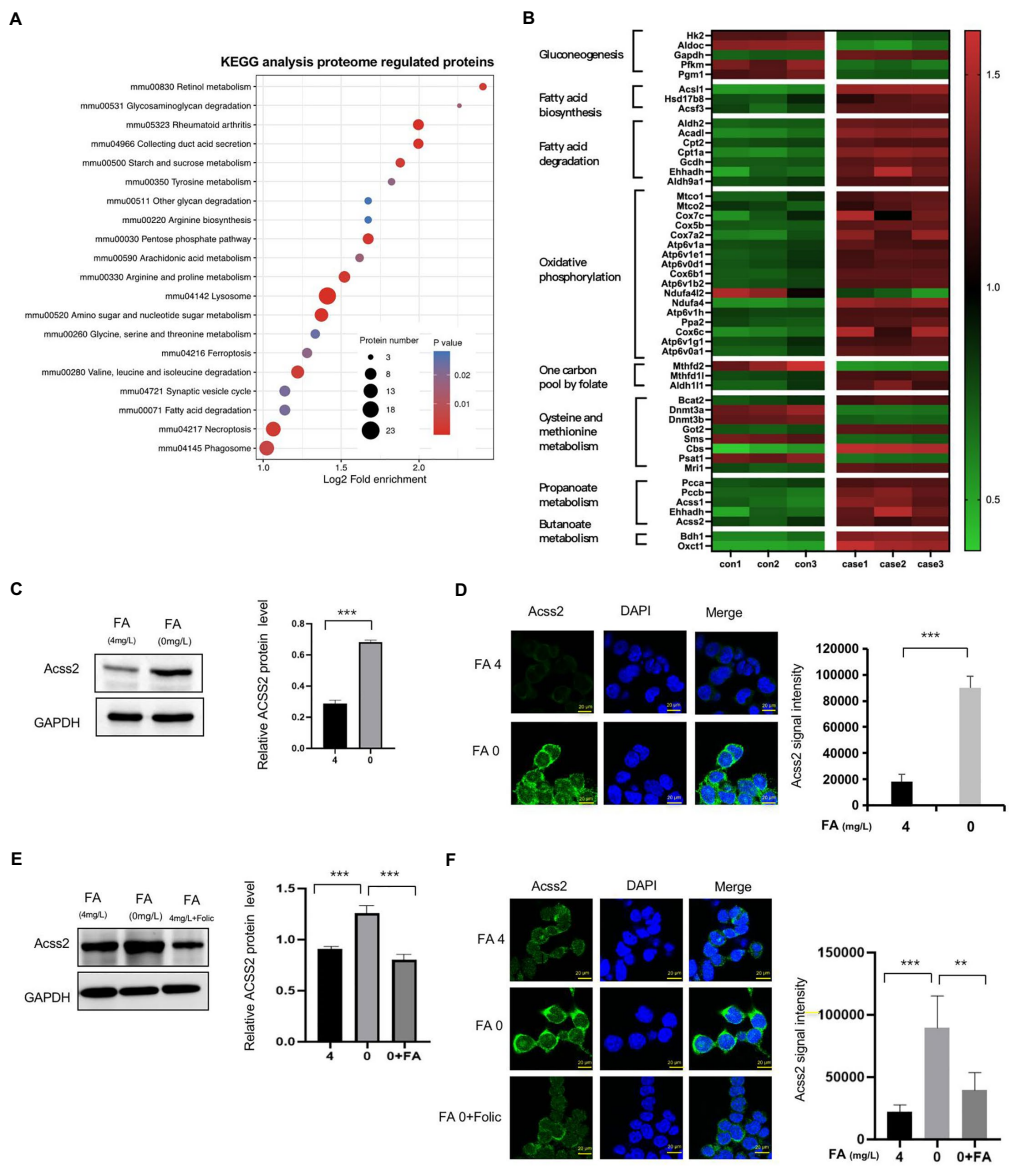
Classification of different proteins by KEGG and expression of ACSS2 under folate deficiency in mESCs. **(A)** Identified DEPs distribution in KEGG. **(B)** Heatmap of different expression proteins in high-confidence proteins shows metabolically distinct patterns in the eight cell states (ANOVA test, FDR *q* < 0.01). DEPs were grouped into clusters based on log_2_ normalized intensity values using hierarchical clustering. **(C)** mESCs were harvested under normal folate versus folate deficiency **(E)** and supplementary folinic acid (50 mg/l) for 24 h analyzed by western blot indicated antibody. Relative ACSS2 protein level was generated by the quantification (ImageJ) of the ACSS2 signal normalized to the GAPDH signal. ^*^p < 0.05, ^**^p < 0.01, ^***^p < 0.001. **(D)** Immunostaining using ACSS2 antibodies revealed discrete foci in mESCs under folate deficiency **(F)** and supplementary folinic acid (50 mg/l) for 24 h. Scale bar: 20 μm. Data are shown as the mean (SD; *n* = 5). Nuclei were stained with DAPI (blue). ^*^p < 0.05, ^**^p < 0.01, ^***^p < 0.001.

In KEGG pathway enrichment, we found that most identified proteins are involved in amino sugar and nucleotide sugar metabolism, pentose phosphate pathway, arginine and proline metabolism, and cysteine methionine metabolism, as shown in Figure 3A and Supplementary Table S4. In addition, higher expression of several metabolism-enriched proteins in folate-free mESCs, including crotonyl-CoA-producing enzymes, appeared to be associated with better prognostic outcomes (log-rank test, *p* < 0.05; Figure 3B). Crotonyl-CoA is an endogenous intermediate metabolite produced during fatty acid oxidation and lysine/tryptophan degradation. Next, we evaluated whether cellular levels of folate influence the expression of ACSS2. Western blot analysis revealed that ACSS2 levels were increased in the absence of folate (0 mg/l) in mESC (Figure 3C). Same results obtain in NE-4C cell (Supplementary Figure S3A). Further immunofluorescence staining analyses in mESCs confirmed that the expression of ACSS2 was induced in folate-free mESCs (Figure 3D, green). Interestingly, remarkable fragments of the crotonyl-CoA-producing enzymes were gathered to the nucleus, suggesting a nuclear role of these enzymes in folate-free mESCs. Next, we supplemented folate-free culture medium with folinic acid (50 mg/l) and added this to mESCs. Western blot results showed that ACSS2 was upregulated under folate deficiency, and this effect was overturned when folinic acid was supplemented (Figure 3E, Line 2 vs. Line 3). Same results obtain in NE-4C cell (Supplementary Figure S3B). It suggested that folinic acid supplementation attenuated increased of the ACSS2 level by folate deficiency. Furthermore, we found that ACSS2 foci were greatly diminished in folinic acid treated cells (Figure 3F). Our results suggest that the ACSS2 induction under folate deficiency in mESCs is closely relevant to folate level. These findings revealed that folate deficiency is particularly related to increment of crotonyl-CoA-producing enzymes *in vivo*.

### 2.4. Global Kcr in folate-free mESCs

**Figure 4:**
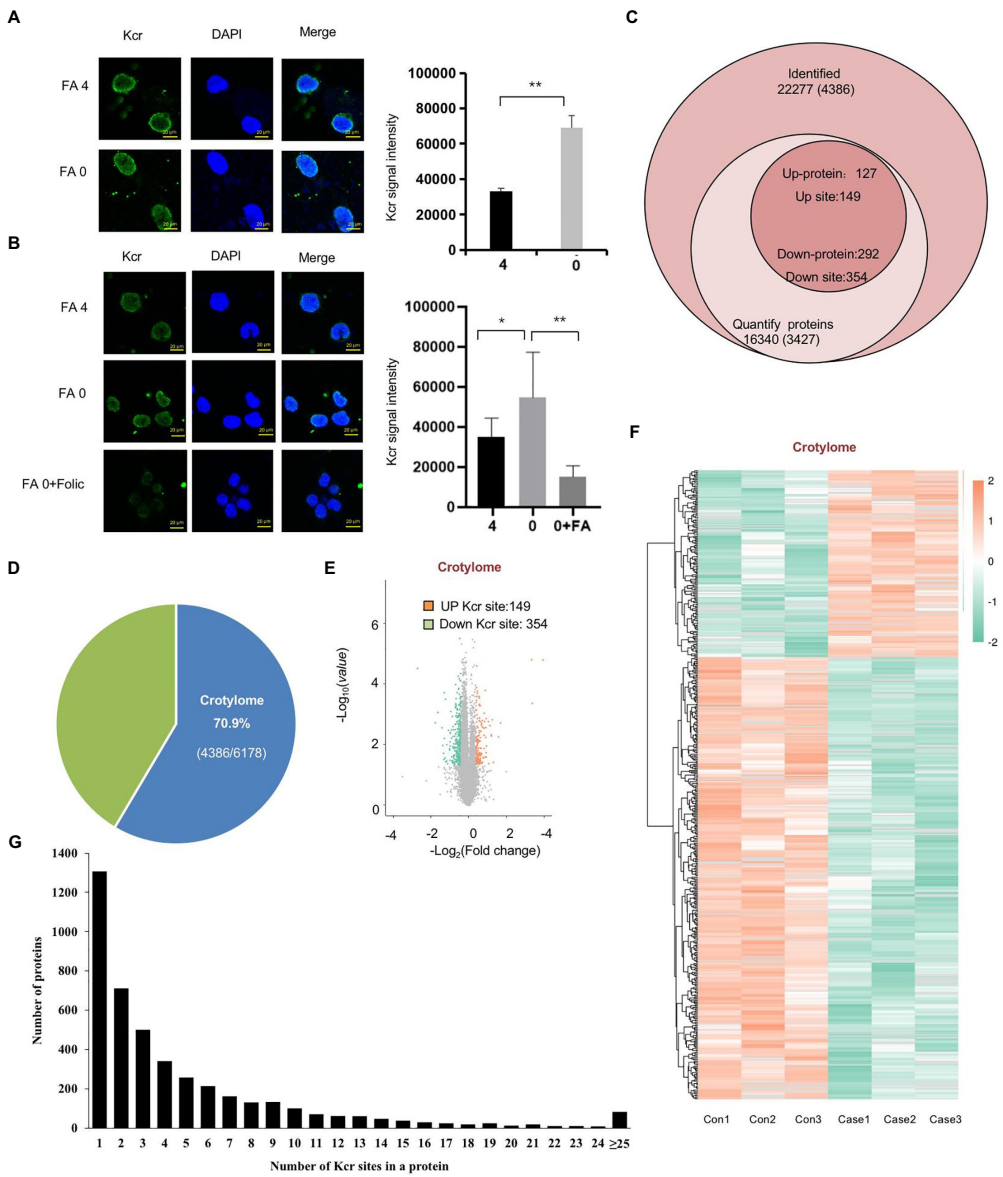
Global crotonylome analysis of folate-free mESCs. **(A)** Immunostaining using anti-crotonlytion (green) antibodies revealed discrete foci in mESCs under folate deficiency **(B)** and supplementary folinic acid (50 mg/l) for 24 h. Scale bar: 20 μm. Data are shown as the mean (SD; *n* = 5). Nuclei were stained with DAPI (blue). ^*^p < 0.05, ^**^p < 0.01, ^***^p < 0.001. **(C)** Global view of identified proteins and crotyl sites. Venn diagram showed the number of proteins and the corresponding identified crotyl sites in brackets. Crotylated proteins with a fold change greater than 1.5 or less than 0.667 and with *p* < 0.05 were considered as significantly up-regulated or down-regulated. **(D)** The relative ratio of crotylated proteins identified in crotylome to that in proteome. **(E)** A volcano plot demonstrating the dual thresholds for total numbers of Kcr sites in normal folate versus folate-free conditions. Each dot represents a single quantified Kcr site. The dots with color in the upper and outer quadrants represent Kcr sites, with green indicating a relative decrease and red indicating a relative increase in abundance (FC ≥ 1.40 or ≤ 0.71, *p* < 0.05). **(F)** Hierarchical clustering heatmap depicting individual samples and Kcr site differences between normal folate versus folate-free mESCs (*n* = 3 per group). **(G)** Statistical map of differential modification sites.

To investigate Kcr altered by folate deficiency, we also found that Kcr was abundant in nuclear foci after exposure to folate deficiency (Figure 4A). Furthermore, we found that Kcr foci were noticeably lessened under folinic acid treatment in cells (Figure 4B). We also found that protein crotonyllysine modification was increased both in mESC and NE-4C cell line in folate deficiency (Supplementary Figures S4A,B). To obtain a global view of the folate deficiency-regulated crotonylome, global crotonylome analysis was performed in folate-free mESCs (*n* = 3) and normal folate mESCs (*n* = 3). High-performance liquid chromatography (HPLC) separation, immunoaffinity enrichment, and mass spectroscopy (MS) detection was applied to investigate Kcr substrates in folate-free mESCs. We identified 22,277 crotyl sites in 16,340 proteins, which constituted 70% of the whole mESC proteome (Figures 4C,D). In addition, 3,427 proteins with 4,386 crotyl sites were quantified (Figure 4C; Supplementary Table S5). Three hundred and fifty-four down-regulated Kcr sites on 292 proteins and 149 upregulated Kcr sites on 127 proteins in total were recognized in folate-free mESCs (fold-change > 1.5; *p* < 0.05) compared with the normal folate group (Figures 4E,F; Supplementary Table S6). Among these Kcr proteins, 1,307 (29.7%) had a single Kcr site, and 1,267 (28.8%) had more than six Kcr sites (Figure 4G; Supplementary Table S7). Together, these results imply that can result into global changes in protein crotylation.

### 2.5. Motif analysis of all identified crotonylated sites

**Figure 5:**
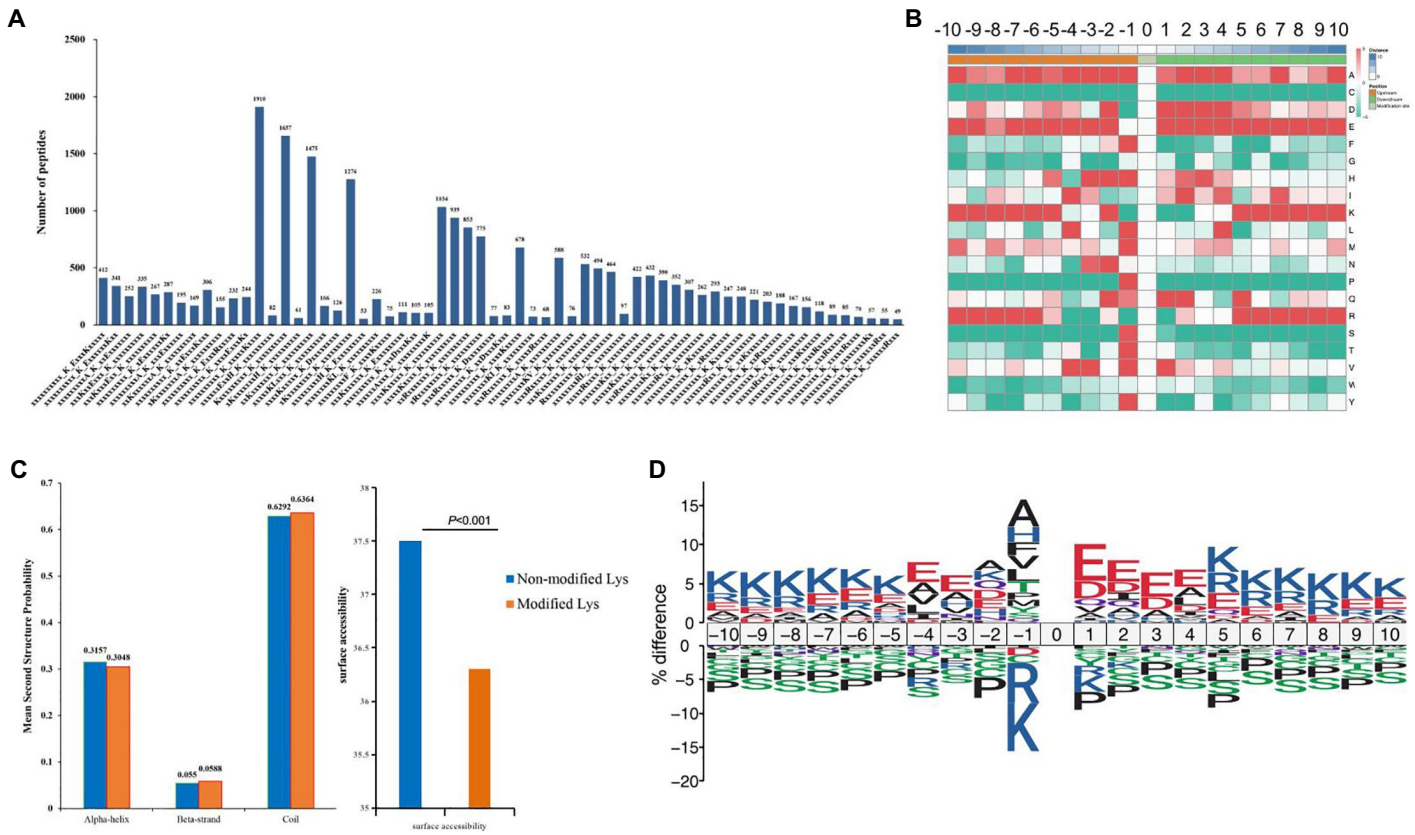
Motif analysis of all identified crotonylated sites in folate-free mESCs. **(A)** Motif statistics of recognized peptides involving crotonylation sites. **(B)** Heatmap showing the different types of amino acids near crotonylated lysines. **(C)** Secondary structure statistics involving identified lysine crotonylation sites. Comparison of various secondary structures (α-helix, β-strand, coil and surface accessibility) involving identified crotonyllysine and all lysine secondary structures. **(D)** IceLogo representation showed sequence preferences for all lysine crotonylation sites.

A total of 22,147 Kcr peptides from all identified peptides with amino acids around the crotonylated lysine from the 210 to 110 positions were analyzed with the Motif-X program (Liu et al., 2021) to determine crotonylated motifs. The crotonylated lysine contexts generated 62 conserved motifs, as shown in Supplementary Table S8. Consistently, analysis using Motif-X algorithms identified xxxxxxxxxx_K_ExxxKxxxxx, xxxxxxxxxx_K_ExxxxxxKxx and xxxxxxxxxA_K_xxExxxxxxx as significantly overrepresented hotspots for Kcr sites, and the data are presented in Figure 5A and Supplementary Table S8. We then investigated the amino acids flanking identified Kcr sites against all mouse background sequences by icelogo (Yu et al., 2019). More negatively charged amino acid (glutamic acid) was detected at Kcr sites (−1 and + 1 positions; Figure 5B; Supplementary Table S9). Structure of Kcr proteins was analyzed by NetSurfP. Analysis showed that about 30.48% of Kcr sites were found, among which 31% were in α-helices, 5.88% were located in β-strands, and the remaining 63.64% were observed in coil structures (Figure 5C). There is no significant difference between the distribution pattern of Kcr and the total protein lysine residues. However, the average surface accessibility of protein Kcr was significantly down-regulated (*p* < 0.001) than total protein lysine residues (Figure 5C). It suggested that Kcr is preferences surface accessibility but not structural preference for Kcr. IceLogo showed predicted amino acid motifs for all Kcr sites (Figure 5D).

### 2.6. Subcellular distribution and GO analysis of significantly up- and down-regulated crotonylated proteins

**Figure 6:**
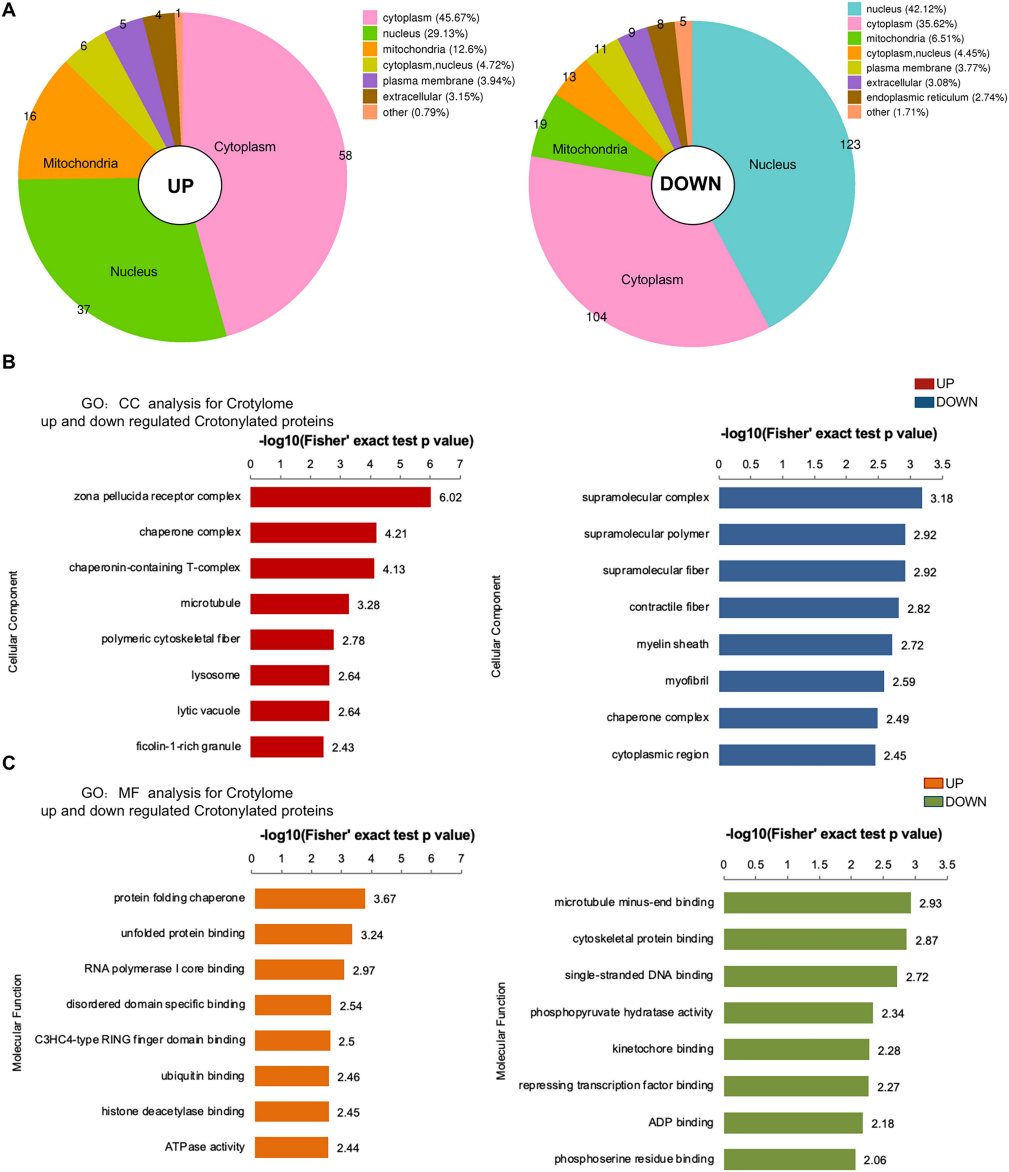
Subcellular location, MF and CC analysis of significantly up- and down-regulated crotylated proteins in folate-free mESCs. **(A)** Subcellular location analysis for significantly up- and down-regulated acetylated proteins, respectively. **(B,C)** Molecular function and cell component annotation for significantly up- and down-regulated crotylated proteins.

To investigate crotonylated proteins in the mESC altered by folate-deficiency, we found 292 proteins were significantly up-regulated and 127 proteins were significantly down-regulated, corresponding to 149 and 354 different crotonylated site, respectively (Figure 4B). There were differences in the subcellular location of up- and down-regulated different crotonylated proteins. As showed in Figure 6A, up-regulated different crotonylated proteins mainly resided in Cytoplasm, while the down-regulated different crotonylated proteins were most abundant in Nucleus (Supplementary Table S10). GO analysis annotation of cell component showed that up- and down-regulated different crotonylated proteins were significantly enriched in zona pellucida receptor complex and supramolecular complex, respectively (Figure 6B). Molecular functions showed that up- and down-regulated different crotonylated proteins were significantly enriched in protein folding chaperone and microtubule minus-end binding, respectively (Figure 6C). This indicates that folate deficiency induced up- and down-regulated crotonylation were mainly involved in different molecular functions processing and cell component in the compartments of cytoplasm and nucleus.

### 2.7. Enrichment clustering protein domain and biological process analysis of the Kcr proteome in folate-free mESCs

**Figure 7:**
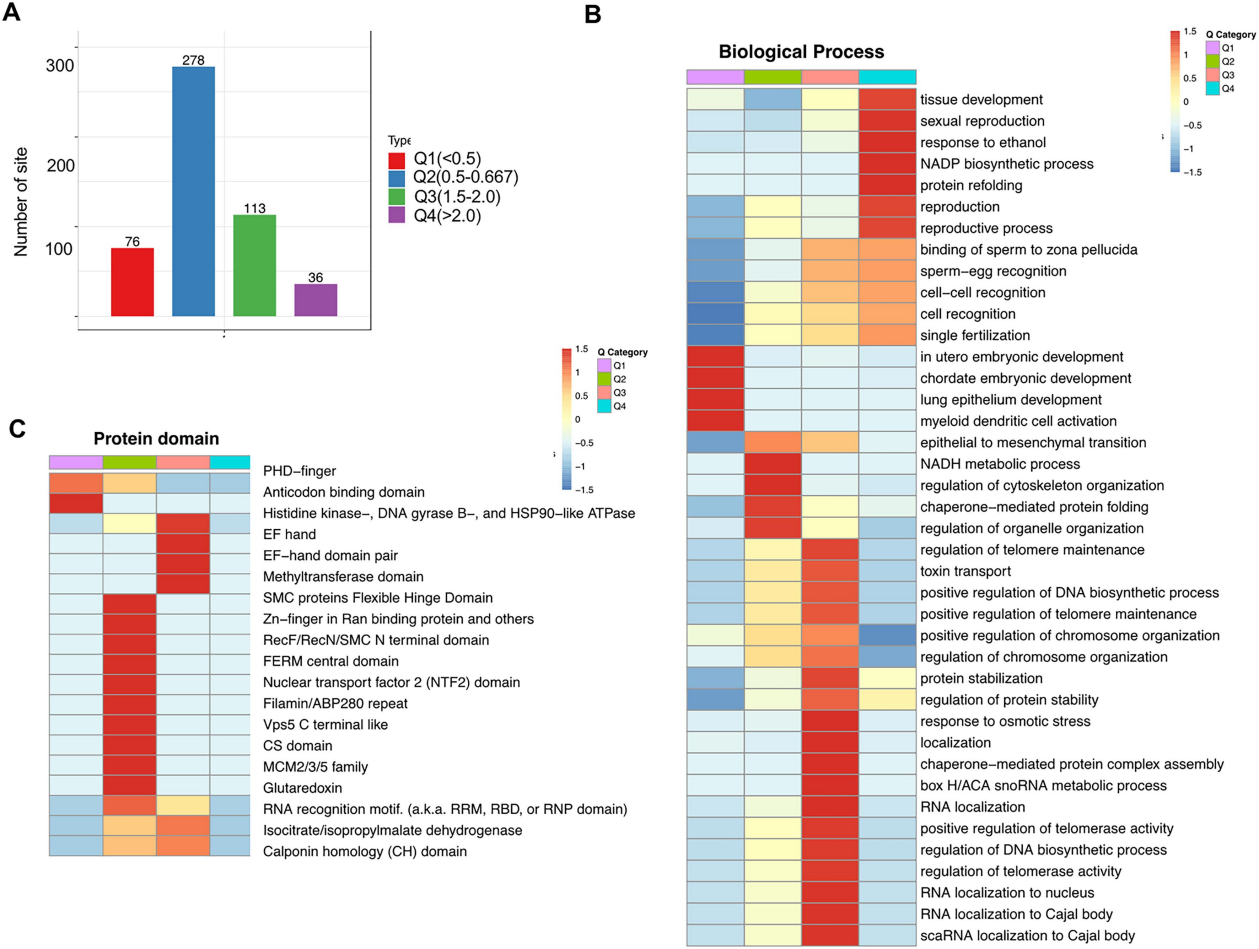
Enrichment analysis of cross-talk between lysine crotonylation and biological processes and protein domains in folate-free mESCs. **(A)** We further divided different Kcr sites into four parts according to the differential expression: Q1 (fold change < 0.5), Q2 (fold change 0.5–0.667), Q3 (fold change 1.5–2.0), and Q4 (fold change > 2). **(B)** Biology process, **(C)** and protein domain. Heatmap showing profiles of all quantifiable Kcr sites in Q1–Q4 normalized according to quantitative proteomics analysis under folate deficiency in mESCs. Fischer’s test *p* values obtained from enrichment analysis were used for hierarchical clustering to combine relevant functions in different Q groups and draw a heatmap. The color block corresponding to the functional description of the enrichment of different Q groups and differentially expressed modified proteins indicated the degree of enrichment. Red represents the enrichment degree; blue indicates the enrichment degree is weak.

To further clarify different crotonylated proteins, we divided them into four categories (Q1–Q4) based on the fold-change values (Figure 7A). Using the GO platform, biological process analysis revealed that Q1 was mainly involved in chordate embryonic development and myeloid dendritic cell activation, Q2 was mainly involved in the regulation of cytoskeleton organization and regulation of organelle organization, Q3 was mainly involved in the regulation of DNA biosynthetic processes and RNA localization to the nucleus, and Q4 was mainly involved in tissue development and NADP biosynthetic processes (Figure 7B; Supplementary Table S11). Protein domain analysis revealed that Q1 Kcr proteins are mainly involved in PHD-finger and anticodon binding domains; Q2 Kcr proteins are mainly involved in SMC protein flexible hinge domains and Zn-finger in Ran-binding proteins; and Q3 Kcr proteins are mainly involved in histidine kinase-, DNA gyrase B-, and HSP90-like ATPase methyltransferase domains (Figure 7C; Supplementary Table S12). These results indicate that folate deficiency can affect multiple biological functions and protein domain by modulating relevant protein crotonylated levels.

### 2.8. Crotonylated lysine in extensive protein network underlies folate-free effects on key cellular metabolism functions

**Figure 8:**
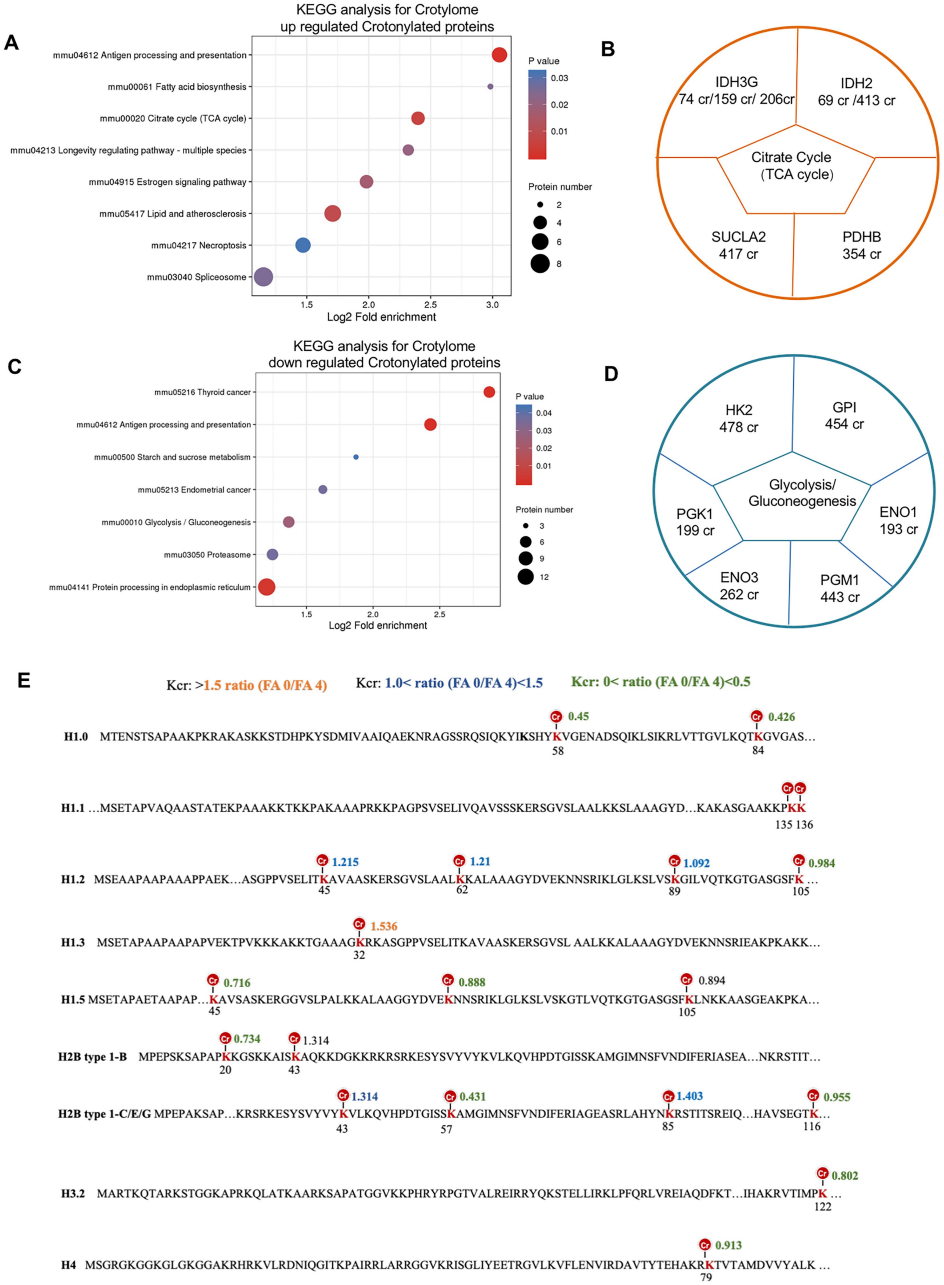
Crotonylated lysine in extensive protein network in folate-free mESCs. **(A,C)** Enrichment analysis of overrepresented KEGG pathways within **(A)** up-regulated or **(C)** down-regulated crotylated proteins in folate-free mESCs (FDR < 0.05). **(B, D)** Selected crotylated sites on significantly enriched metabolism pathways and quantification of exemplary crotylated sites that were **(B)** upregulated or **(D)** downregulated in folate-free mESCs. **(E)** Histone Kcr sites identified and quantitative crotonylome analysis in normal folate versus folate-free mESCs. Histone lysine sites with different changes in the Kcr level are color-coded.

To learn more about the functions of the crotonylated protein, we performed pathway and KEGG analysis. For the upregulated and downregulated sites, the most enriched metabolism pathways shared by these crotonylated proteins were related to citrate cycle (TCA cycle) and glycolysis/gluconeogenesis, respectively (Figures 8A,C). Examples are shown and quantified in Figure 8B. Proteins involved in citrate cycle (TCA cycle) whose Crotonylation was upregulated in folate-free included IDH3G (IDH3G 74 cr, IDH3G 159 cr and IDH3G 206 cr) and IDH2 (IDH 69 cr, IDH2 413 cr) linked to alternative TCA cycle regulation. Down-regulated sites crotonylation of Glycolysis/Gluconeogenesis regulators included a diverse set of proteins with HK2 (HK2 478cr) and PGK1 (PGK1 199cr; Figure 8D). The critical role of histone modification is epigenetics control of gene activation and silencing. A picture of the canonical histones and histone variants with crotonylation was manifested (Figure 8E). Twenty crotonylated core histone lysine sites were identified and quantified in total, and six sites showed more increases in crotonylation in folate-free mESCs (Figure 8E; Supplementary Table S14). For example, we detected a 1.5-fold increase in H1.3K32cr in folate-free mESCs (Figure 8E). All the other sites exhibited slight changes in Kcr levels (Figure 8E). These results further support the proposition that folate deficiency positively regulates histone Kcr in cultured cells. We also summarized the histone crotylation sites (Folmes et al., 2011) found on classical and variant histones in Figure 8E. Among them, crotonylated modifications were detected in H3K122, H4K79, H2B type 1-B K20, H2B type 1-B K43, H2B type 1-C/E/G K57, K85, and K116, which play an important role in epigenetic regulation. Acetylation sites associated with transcriptional activation included H3K122ac and H4K79ac (Mitchell, 2005). These double or higher-order modifications imply a more complicated level of gene regulation through cross-talk acylation epigenetic mechanisms. Our research reveals the critical regulatory roles of Kcr in glycolysis/gluconeogenesis, and the citrate cycle (TCA cycle) metabolism in folate-free mESCs.

## 3. Discussion

In this study, 22,277 Kcr sites were recognized in 16,340 proteins through a quantitative proteomics approach. It expands the understanding of the Kcr proteome and provides a comprehensive mESC landscape of Kcr. In folate-free mESCs, we identified 354 down-regulated Kcr sites on 292 proteins and 149 up-regulated Kcr sites on 127 proteins (fold-change > 1.5; *p* < 0.05). The crotonylated lysine contexts generated 62 conserved motifs, as shown in Supplementary Table S9. Consistently, analysis using Motif-X algorithms identified xxxxxxxxxx_K_ExxxKxxxxx, xxxxxxxxxx_K_ExxxxxxKxx and xxxxxxxxxA_K_xxExxxxxxx as significantly overrepresented hotspots for Kcr sites, and the data were shown in Figure 5A. We identified fatty acid oxidation enzymes, including ACSS2, as key crotonyl-CoA producers under folate deficiency. We also showed that ACSS2 and Kcr were elevated in mESCs under folate deficiency but attenuated by folinic acid supplementation. Folate deficiency specifically induced intracellular short-chain fatty acids, increased ACSS2 and Kcr, and involved in NTDs (Figure 1). Therefore, our data indicate that folate deficiency contributes to the onset of NTDs by altering ACSS2 and demonstrate that Kcr may be a metabolic-sensitive protein modification. Accordingly, crotonylation might be important in the development of the neural tube and functions of the fetal brain with a high demand for folate and/or lysine or tryptophan metabolism.

Neural tube defects (NTDs) are caused by the genetics and environmental factors (Wallingford et al., 2013). Many studies support the importance of maternal nutrition in the proper development of offspring and suggest that epigenetics is imperative for proper NTC. Folic acid deficiency during pregnancy is an important dietary factor in the development of NTDs. It has become an international consensus that the use of folic acid-fortified foods during pregnancy reduces the incidence of NTDs (Wilde et al., 2014). In addition, carbon metabolism has received increased attention because folic acid is involved in carbon metabolism. Maternal diabetes is considered a risk factor for a variety of congenital birth defects, including NTDs (Zhang T. et al., 2013). By studying the molecular mechanism of NTDs caused by diabetes, several measures have been proposed to protect developing embryos. For example, folate supplementation in diabetic rats can reduce the incidence of NTDs (Gäreskog et al., 2006). In addition, folic acid reduced the risk of NTDs in gestational diabetes and had a greater protective effect on anencephaly than spina bifida (Correa et al., 2012). Recently, our studies have also shown that abnormal homocysteine metabolism is related to the occurrence of NTDs (Zhang et al., 2018). Maternal homocysteine abnormalities during pregnancy are associated with an increased risk of NTDs in offspring. In this study, we detected increased of short-chain fatty acids in the brain of mice with NTDs using an LC–MS/MS platform under low maternal serum levels of folate. The results showed that butyric acid was significantly increased by 100-fold, and isovaleric acid was increased by 3-fold. However, no significant changes in acetic acid, propionic acid, pentanoic acid, or hexanoic acid were observed (Figure 1B). Short-chain fatty acids are likely a physiologically relevant source of acyl-CoA within cells. ACSS2 has been implicated as the enzyme that generates crotonyl-CoA from crotonate. The depletion of ACSS2 leads to reduced levels of histone Kcr, which may potentially increase the synthesis of acyl-CoAs from endogenous short-chain fatty acids.

The development of the neural tube occurs during neural induction when the flat layer of neural epithelial cells surrounded by epidermal cells folds and closes, forming the neural tube covered by the surface ectoderm. NTC occurs progressively, beginning in the caudal/lateral notochord of the rboencephalin and then passing through the head and down the spinal cord during the third to 4th weeks of gestation in a coordinated, dynamic process (Aguilar et al., 2012). Neural tube development is a complex multi-step process involving gene–gene, gene–environment, and gene-nutrition interactions. It is strictly controlled by genes and environmental factors (Harris, 2009). The nervous system is one of the most complex systems in the body. During neural tube development, if the interference of genetic or environmental factors exceeds the compensatory capacity of the embryo, NTDs may occur (Harris and Juriloff, 2007). Previous studies have identified enzymes that specifically induce the production of crotonyl-CoA in endoderm cells and mouse embryos during *in vitro* human embryonic stem cell differentiation (Fang et al., 2021). This suggests that crotonylation is a key mechanism mediating the effects of these crotonyl-CoA-producing enzymes on endoderm differentiation. Our data showed that Kcr was increased in the cranial neural tube of embryos with folate deficiency-induced NTDs compared with that in wild-type controls, suggesting that open NTDs involve the upregulation of ACSS2 (Figure 1D). Recent studies showed that folate deficiency induces the expression of genes involved in lipid metabolism(Pooya et al., 2012). It revealed that expression of lipid biosynthetic genes, including acetyl CoA synthetase long-chain family member 1, lipin 1, diacyl-glycerol O-acyltransferase 2, and ATP citrate lyase were increased, suggesting increased lipid biosynthesis under folate deficiency (Fukuoka and Kubota, 2018). The expression of CPT1A, carnitine O-octanoyltransferase, and acetyl-coenzyme A dehydrogenase was increased in folate-free mice (Champier et al., 2012). In this study, we showed that folate deficiency affected ACSS2 in mESCs (Figures 3C,D). It is important to explore the relationship between folate and epigenetic changes affecting essential fatty acid metabolism. The *Fads2* gene encodes delta-6 fatty acid desaturase (D6D; Devlin et al., 2007). It was found that hypermethylation in the promoter region of *Fads2* was reduced *Fads2* mRNA expression and decreased D6D activity in cystathionine-β-synthase heterozygous mice (Devlin et al., 2007). PPARc promotes lipid storage (Gavrilova et al., 2003). Studies have shown that folic acid supplementation *in utero*, postpartum, or after weaning can regulate the methylation of the PPAR promoter in rat livers (Sie et al., 2013). We also found that folic acid supplementation attenuates ACSS2 induced by folate deficiency. One potential mechanism is that promoter methylation inhibits gene expression and increases the expression of ACSS2 in response to folate supplementation. It is important to clarify the detailed regulatory mechanisms in this aspect in future investigations.

ACSS1, ACSS2, and EHHADH are related to the key enzymes that produce crotonyl CoA (Bhala et al., 1995). We found that these factors are regulated by folic acid, indicating the existence of a feedback loop between cellular pools of acetyl-CoA/short-chain acyl-CoA and protein Kcr in metabolic pathways. Clarifying the detailed regulatory mechanisms is essential in the future. Kcr, Kac, and Kbu have similar human embryonic stem cell structures (Satija et al., 2015), but unlike Kac and Kbu, Kcr provides transcriptional specificity for mesoderm/endoderm lineage commitment. It is necessary to induce the expression of crotonyl-CoA-producing enzymes and determine the underlying molecular mechanisms that regulate Kcr during neural tube development. Crotonyl-CoA is a precursor of Kcr and is usually generated by fatty acid oxidation. Recently, a study showed that short-chain fatty acid metabolites and their corresponding CoAs produced by gut microbes promote histone Kcr and affect gene transcription in the host colon (Fellows et al., 2018). This illustrates that a close functional link between cellular metabolism and protein Kcr regulation is predictable in different biological contexts. We found that identified Kcr proteins are associated with multiple KEGGs, such as the key regulatory roles of Kcr proteins HK2, PGK1, IDH2, IDH3G in glycolysis/gluconeogenesis and citrate cycle (TCA cycle) metabolism in folate-free mESCs (Figures 8A–D). After exposure to folate deficiency, we showed that crotonylation lysines were abundant in the nucleus. Furthermore, we found that Kcr foci were significantly reduced in cells treated with folinic acid (Figures 4A,B). The staining of total Kcr was increased in NTD mice compared with that in normal tissues (Figure 1D).

We also summarized the histone crotylation sites found on classical and variant histones under folate deficiency in mESCs (Figure 8E). While mapping total of 20 crotonylated core histone lysine sites, a seven histone tails were simultaneously modified by other diverse acylation modifications, such as crotonylated sites H2BK20/43/57/85, H3K122 and H3K79. Crotonylated modifications were detected in H3K122, which play an important role in acetylation sites during transcriptional activation. Several studies also showed that histone Kcr sites play important in different physiological functions. H4K77cr and H4K91cr were found in endoderm differentiation, suggesting function in modulating DNA/nucleosome dynamics and stability (Bowman and Poirier, 2015; Lawrence et al., 2016). Crotonylated modifications were detected in H3K122 and H4K79, which play an important role in acetylation sites during transcriptional activation. H3K9cr suppressed the expression of growth-related genes. H3K18cr enhanced the expression of genes related to myocardial hypertrophy (Gowans et al., 2019). H3K27cr are important for the proper differentiation of round spermatids into final sperm (Crespo et al., 2020). Further investigations determined the role of histone crotonylation and identified NTD-related genes under folate deficiency conditions.

In conclusion, our study is the first systematic analysis of Kcr substrates and provides the largest crotonylome dataset in folate-free mESCs to date. We further elucidated that induced ACSS2 leads to Kcr upregulation associated with NTDs. Our improved understanding of the histone modification code of complex proteomes provides clues for future neurodevelopment research, which may help prevent birth defects.

## 4. Materials and methods

### 4.1. Animals

Male and female C57BL/6 mice (6–8 weeks, 18–20 g) were purchased from the Jinmuyang Animal Laboratory. Sexually matured mice were mated overnight, and were separated and transferred to different cages at 8:00 am the next day, which was recorded as E0.5. On E7.5, female mice with vaginal plug detected were intraperitoneal injected with 1.5 mg/kg (body weight) methotrexate (MTX, Sigma, United States, cat#A6770-10MG). On E13.5, mice were sacrificed under euthanasia. All routines to handle the animals complied with the care of laboratory animals’ guidelines. Other detail as produced in previously study (Pei et al., 2019).

### 4.2. Immunohistochemical staining

We sectioned the embedded paraffin sections and then performed immunohistochemical staining with primary antibodies for Kcr, ACSS2, acyl-CoA dehydrogenase (ACADS), and peroxisomal acyl-coenzyme A oxidase 1 (ACOX1). We incubated tissues with Kcr (1:100 dilution; Jingjie China, cat# PTM-501) ACSS2 (1:100 dilution; Abcam, United Kingdom, cat#ab133664), ACADS (Anti-ACADS/SCAD, 1:100 dilution; Abcam, United Kingdom, cat#ab156571), and ACOX1 (Anti-ACOX1/AOX, 1:100 dilution; Abcam, United Kingdom, cat# ab184032) at 4°C for 12 h (Fang et al., 2021). Gray-scale analysis (ZEN 2012 ZEISS COMPANY) was used to analyze the levels of Kcr, ACSS2, ACADS, and ACOX1. We ensured quality control of each lot of slides.

### 4.3. Targeted metabolomic analysis.

UPLC-ESI-MS/MS analysis was performed to qualitative and quantitative detection of SCFAs with 30 mg brain samples. Metabolite quantification was analyzed using MRM mode of triple quadrupole mass spectrometry. Data obtained from Mass spectrometry in different samples were chromatographic peaks and the peak areas were integrated on all chromatographic peaks. The peak areas denotes the relevant metabolites (He et al., 2022).

### 4.4. Cell culture and conditions

Cell culture medium was prepared under aseptic conditions with 0.1 mM β-mercaptoethanol (Invitrogen, Carlsbad, CA, United States, cat#21985023), 0.1 mM non-essential amino acids (Invitrogen, Carlsbad, CA, United States, cat#11140050), 0.1 mM glutamate (Invitrogen, Carlsbad, CA, United States, cat#35050061), 15% fetal bovine serum (Gibco, United States, cat#10099141), 4 mg/l folate (Sigma-Aldrich, cat#F8758-5G) and 1,000 U/ml mouse leukemia inhibitory factor (Millipore, Billerica, United States, cat#ESG1107). Folate treatments of ESC cultures as produced in our previously studies (Chang et al., 2013; Zhang Q. et al., 2013).

### 4.5. Liquid chromatography tandem mass spectrometry (LC–MS/MS)

Peptides were dissolved in liquid chromatographic mobile phase A containing 0.1% formic acid and 2% acetonitrile and separated with Bruker NanoElute 2 high-performance nanoflow liquid chromatography system, then solution went through a capillary ionization system, and analyzed by timsTOF Pro mass spectrometry (MS). Mobile phase B was 100% acetonitrile solution with 0.1% formic acid. The liquid phase Gradients of the liquid phase were as follows: 0–70 min, 3–22% B; 70–85 min, 22–33% B; 85–87 min, 33–80% B; and 87–90 min, 80% B. The flow rate was set to 300 nl/min. Voltage of the ion source was 1.5 kV. Parent ions and their secondary fragments of the targets were detected in high-resolution time-of-flight mass spectrometer. The scanning range of secondary mass spectrometry was *m/z* 100–1,700. The secondary spectra were collected in a scan mode termed the parallel cumulative serial fragmentation (PASEF) mode straight after a first-order MS collection. Charge numbers of the parent ions in the secondary spectra were ranged from 0 to 5. Collection repeated ten times. To avoid repeated scanning of parent ions, the dynamic exclusion time was set to 30 s. Data were processed in MaxQuant with the integrated Andromeda search engine (version 1.5). False discovery rate (FDR) thresholds for protein, peptide, and modification sites were specified at 1% for targets (protein, peptide, and modification sites). Cutoffs for significant fold changes between cells by histone crotonylation were set as quantification ratios above 1.5 or below 0.67 (Yang et al., 2021; Zhang et al., 2022).

### 4.6. Database search

The original mass spectrometry data were imported into the search database software, and the corresponding analysis parameters were set according to the experimental scheme. The secondary MS data was processed in Maxquant (V1.6.15.0). Trypsin/P was given to the digestion mode and only two missing cleavages were allowed. The smallest length of the peptide was limited to seven and the largest modification number of peptides was five. The mass error tolerance of primary parent ions in the First search and Main Search was set at 20 PPM and 20 PPM, respectively, and that of secondary fragment ions was set at 20 PPM. Fixed modification was positioned for alkylation of cysteine carbamidomethyl, variable modification for oxidation of methionine and n-terminal acetylation. The FDR value was 1% for protein identification and PSM identification. The database underwent further filtering to achieve high quality and trustworthy results. The FDR values of the spectrogram, peptide, and protein identification were all less than 0.01. Identified protein had at least one unique peptide (Yang et al., 2021; Zhang et al., 2022).

### 4.7. Bioinformatics analysis

Data bank successfully helped us to annotate Gene Ontology (GO) regarding cell compositions, biological processes, and molecular functions. GO annotation is to annotate and analyze identified proteins using eggnog-Mapper software (V2.0). The software is based on the EggNOG database, and the latest version is the fifth version, covering 5,090 organisms (477 eukaryotes, 4,445 representative bacteria and 168 archaea) and 2,502 viruses. Here, the GO IDS in the annotation results of each protein are extracted, and then the proteins are functionally classified in line with cellular components, molecular functions and biological processes. Protein pathways were annotated based on KEGG pathway database, and basic local alignment search tool (BLAST) comparison (BLASTP, E value ≤ 1E-4) was performed to identify proteins., The result of the highest score was chosen for annotation to compare results in each sequence. InterProScan software is used to annotate protein domain of recognized modified proteins. We used WoLF PSORT to annotate the subcellular location of the identified proteins. We considered categories with a *p* value of 0.05 to be significant. We considered the sequence context to be the motif of the crotonylated peptide when the characteristic sequence number exceeded 20 and the *p* value was 0.000001. The sequence model composed of specific amino acids was analyzed by Motif-x (10 amino acids downstream and upstream of crotonylation sites). Once protein enrichment was completed, function *x* = 2log_10_(*p* value) ran to convert the *p* value and one-way hierarchical clustering to category (Qian et al., 2022).

### 4.8. Tandem mass tagging proteomic analysis

We obtained support from Jingjie PTM Biolabs (Hangzhou, China) for Tandem mass tagging-based proteomics analysis(Liu et al., 2021). The detailed procedures can be found in the Supplementary Data. Enrichment of differentially modified proteins relevant to all identified proteins for each category of GO analysis, including cellular compartment, biological process, and molecular function was detected in two-tailed Fisher’s exact test. We considered GO terms significant at a corrected *p* < 0.05 and used DAVID 6.8 to perform GO term enrichment(Qian et al., 2022).

### 4.9. Western blotting

Proteins extractions were separated on sodium dodecylsulphate (SDS) polyacrylamide gel through electrophoresis and transferred to a nitrocellulose membranes. The membrane was soaked in 5% milk to block the non-specific proteins for 5 min and incubated in the primary antibodies at 4°C overnight, including a mouse anti-ACSS2 monoclonal antibody (1:1,000, Abcam, United Kingdom, cat#ab133664) and mouse anti-Kcr monoclonal antibody (1:1,000, Jingjie, China, cat#PTM-501). Then, we incubated the membranes with the corresponding secondary anti-rabbit horseradish peroxidase-conjugated antibody (1:5,000, Abcam, United Kingdom, cat#ab205718) for 1 h at room temperature. Finally, we visualized the membrane using SuperSignal West Pico Chemiluminescence Substrate (Thermo, United States, cat#34580) and quantified protein bands using Quantity One software.

### 4.10. Fluorescence analysis

Cells were planted in 35 mm culture dish and fixed in 4% paraformaldehyde. After fixation, 0.1% Triton X-100 (PBS) was used as permeabilizing reagents. Primary antibodies, including Kcr (1:100; Jing Jie, China, cat#PTM-501) and ACSS2 (1:200; Abcam, United Kingdom, cat#ab133664), were added to cells and incubated overnight at 4°C. Next, cells were incubated with TRITC-conjugated or FITC-conjugated secondary antibody (dilution 1:200; Zhong Shan Jin Qiao, China, cat#ZF-0511) at 37°C for 1 h. Nuclei were stained with DAPI (Solarbio, China, cat#C0065), and images were visualized with a Zeiss LSM 510 Element inverted confocal microscope.

### 4.11. Statistical analysis

All experiments with our final conclusion were completed in triplicate, and the data were presented as the mean ± standard deviation. The Student’s *t*-test was performed to give statistical significance. *p* value of < 0.05 was considered statistically significant and presented as ^*^p< 0.05, ^**^p< 0.01 or ^***^p< 0.001.

## Data availability statement

The datasets presented in this study can be found in online repositories. The names of the repository/repositories and accession number(s) can be found in the article/Supplementary material.

## Ethics statement

The study was conducted according to the guidelines of the management and utility of experimental animals and approved by the Capital Institute of Pediatrics Ethics Committee (DWLL2021011), and all animal manipulations were strictly performed following the relevant laws of China. Human samples were collected and analyzed in accordance with the Capital Institute of Pediatrics Ethics Committee approval (SHERLLM2021024).

## Author contributions

SW conceived and designed this study. SW, YZ, and PP participated in laboratory work. SW, YZ, FL, and XH performed the data analysis. SW and PP participated in the writing of the manuscript. SW and TZ participated in advising and revising the manuscript critically. All authors contributed to the article and approved the submitted version.

## Funding

This work was supported by the National Natural Science Foundation Projects (82071690 and 81971390), Research Foundation of Capital Institute of Pediatrics (FX-2020-05 and CXYJ-2-21-09), Beijing Hospitals Authority’s Ascent Plan (DFL20221102), Public Service Development and Reform Pilot Project of Beijing Medical Research Institute (BMR2021-3).

## Conflict of interest

The authors declare that the research was conducted in the absence of any commercial or financial relationships that could be construed as a potential conflict of interest.

The reviewer QX declared a shared parent affiliation with the author FL to the handling editor at the time of review.

## Publisher’s note

All claims expressed in this article are solely those of the authors and do not necessarily represent those of their affiliated organizations, or those of the publisher, the editors and the reviewers. Any product that may be evaluated in this article, or claim that may be made by its manufacturer, is not guaranteed or endorsed by the publisher.
