# Supplementary material for: Folate-deficiency induced acyl-CoA synthetase short-chain family member 2 increases lysine crotonylome involved in neural tube defects
Source: Front Mol Neurosci. 2023 Jan 20;15:1064509. doi: 10.3389/fnmol.2022.1064509 (PMC9895841; doi:10.3389/fnmol.2022.1064509)

Figure S1

A

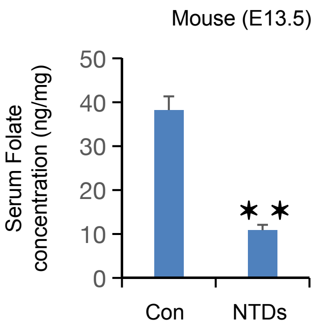

B

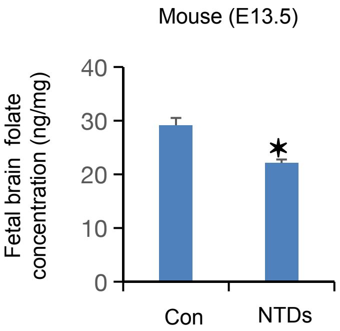

C

	Folate Concentration(ng/mg)					
Group	CON	Gestational/ Weeks	Gender	NTD	Gender	Gestational/ Weeks
1	0.223466667	18	Male	0.057666667	Male	28
2	0.204933333	16	Male	0.026667	Male	35
3	0.2632	15	Female	0.060133333	Male	28
4	0.2264	20	Male	0.066933333	Female	32
5	0.188533333	21	Male	0.065133333	Female	34
6	0.15918396	16	Male	0.068933333	Female	25

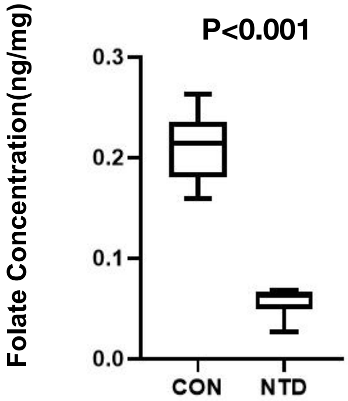

D

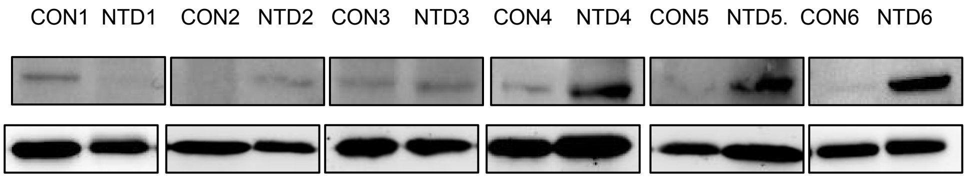

E

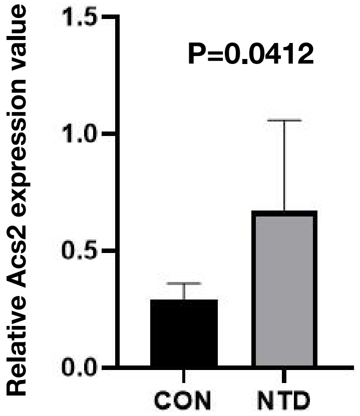

Figure S2

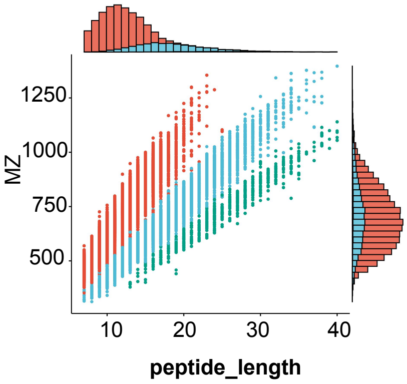

Figure S3

A

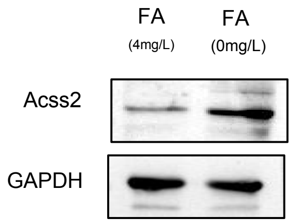

B

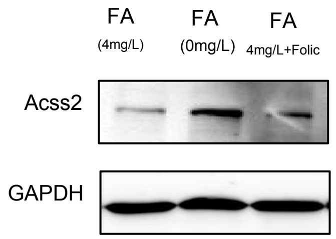

Figure S4

A

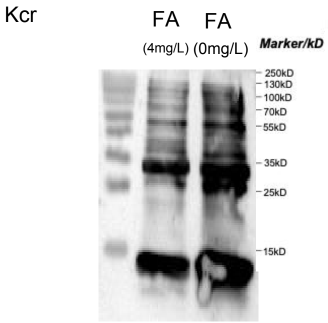

B

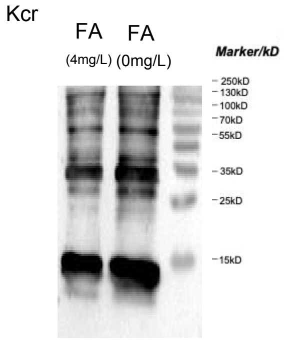

Scan blot

Figure S5

Fig 2C
ACSS2

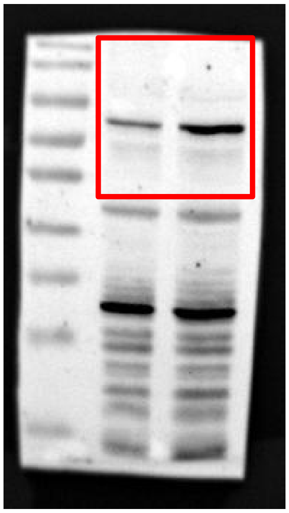

Fig 2C
GAPDH

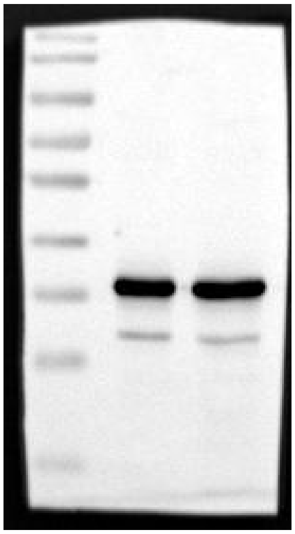

Fig 2D
ACSS2

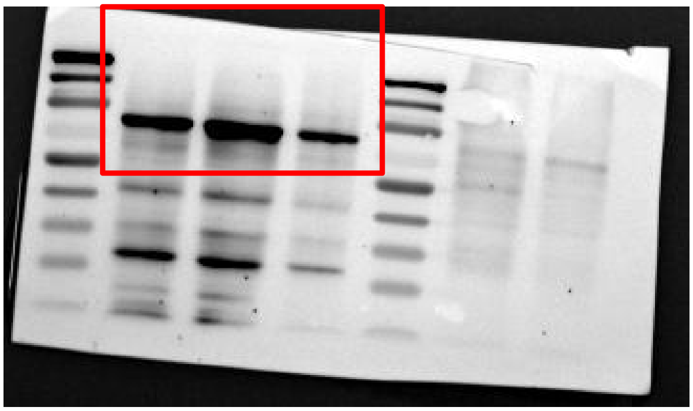

Fig 2D
GAPDH

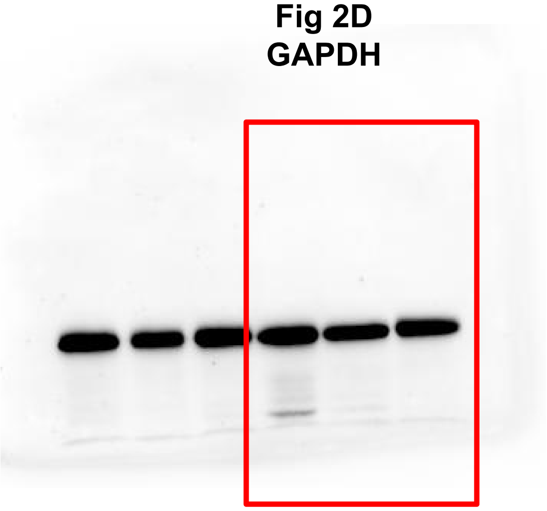

Fig S1 D
ACSS2

CON1 NTD1 CON4 NTD4

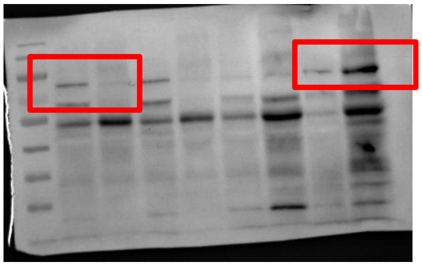

Fig S1 D
ACSS2

CON43 NTD3

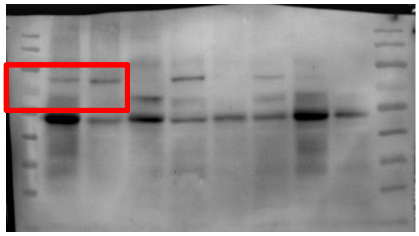

Fig S1 D
GAPDH

CON1 NTD1 CON4 NTD4

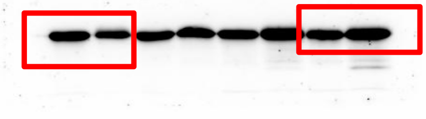

Fig S1 D
GAPDH

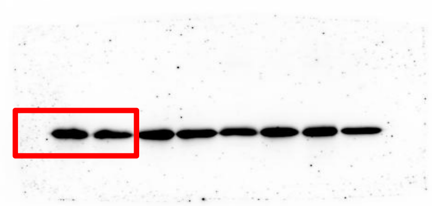

Fig S1 D
ACSS2

CON2 NTD2. CON5 NTD5

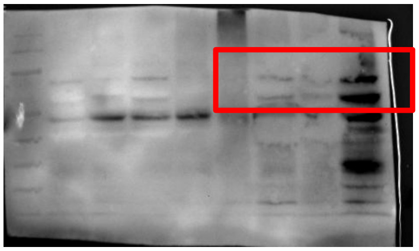

Fig S1 D
ACSS2

CON46 NTD6

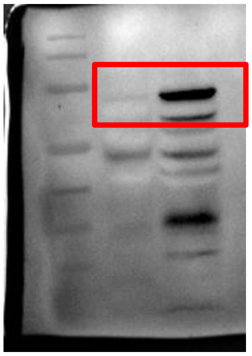

Fig S1 D
GAPDH

CON2 NTD2 CON5 NTD5

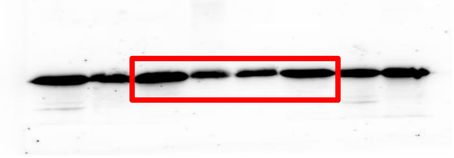

Fig S1 D
GAPDH

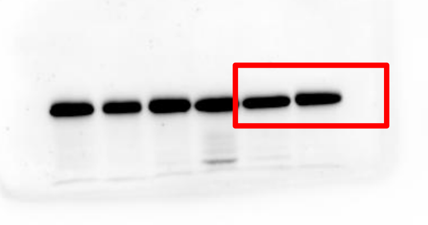

Fig S3A
ACSS2

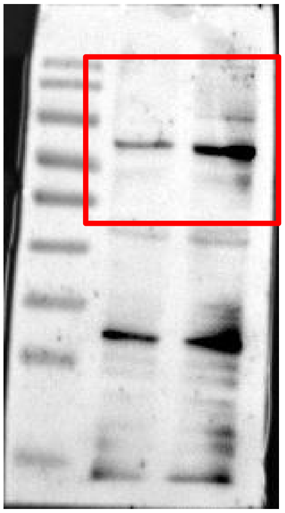

Fig S3A
GAPDH

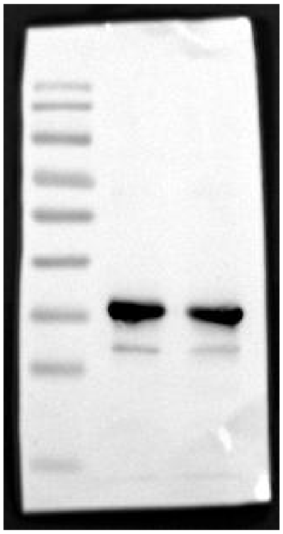

Fig S3 B
ACSS2

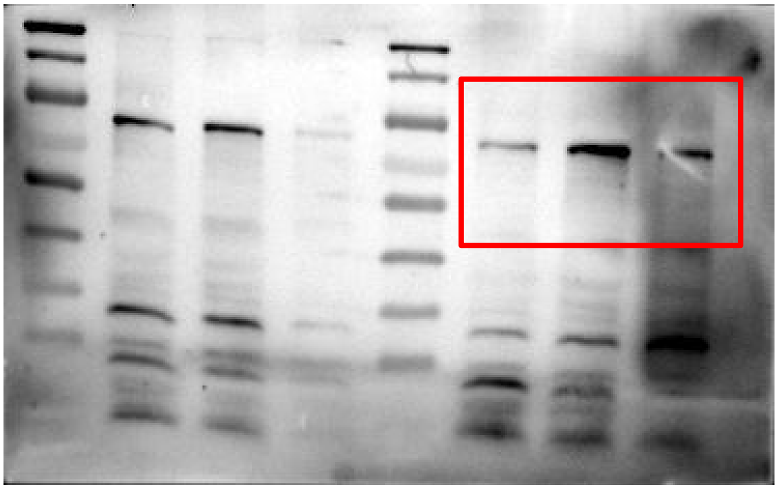

Fig S3B
GAPDH

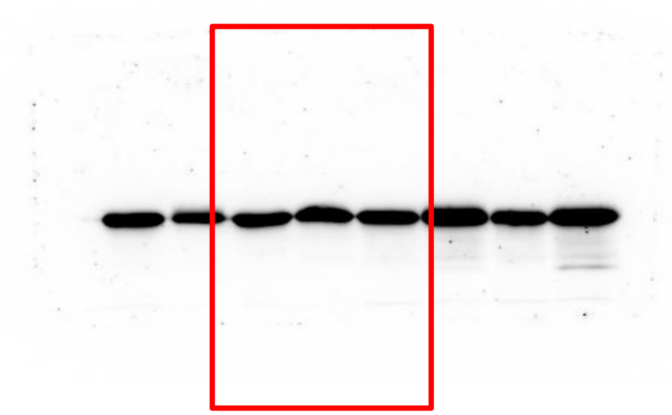

Supplement: Supplementary file 1 [file Image_1.pdf]
