# Supplementary material for: Folate-deficiency induced acyl-CoA synthetase short-chain family member 2 increases lysine crotonylome involved in neural tube defects
Source: Front Mol Neurosci. 2023 Jan 20;15:1064509. doi: 10.3389/fnmol.2022.1064509 (PMC9895841; doi:10.3389/fnmol.2022.1064509)
Supplement: Supplementary file 2 [file Table_1.docx]

**Supp Materials and methods**

**protein extraction, and trypsin digestion**

Cells were thoroughly ground with liquid nitrogen and then dissolved in lysis buffer (1% Triton X-100, 1% pro- tease inhibitors, 2mM EDTA, 3mM Trichostatin A [TSA], 8 M urea, 10mM dithiothreitol [DTT], 50 mM nicotinamide [NAM]). It was then placed on ice to cool and treated with high-intensity ultrasound processor (scientz, China) for 3 times (30s at 2-min intervals). The above treated lysates were precipitated at 12000 g for 1 hour, and then stored in a refrigerator at 4 ° C. The proteins collected were mixed with 15% TCA and then overnight at 220 ° C. The precipitate was washed with cold acetone three times. The protein was re dissolved in 8 M urea, and the protein concentration was determined with a dioctenic acid (BCA) kit (lot number 23225, Thermo Scientific, USA).

Equal amounts of protein from each sample were enzymatically hydrolyzed and the volume adjusted to match the lysate. TCA at a final concentration of 20% was slowly added, vortexed, and allowed to settle for 2h at 4°C. Centrifuge at 4500 g for 5 min, discard the supernatant, and wash the pellet 2-3 times with pre-cooled acetone. After drying the precipitation, add TEAB at a final concentration of 200 mM, disintegrate the precipitation by sonication, add trypsin (protease: protein, m/m) at a ratio of 1:50, and perform enzymatic dissociation overnight. Dithiothreitol (DTT) was added to a final concentration of 5mM and reduced at 56 °C for 30 min. Iodide acetamide (IAA) was then added to a final concentration of 11 mM and incubated for 15 min at room temperature.

**Modification of enrichment**

The peptide was dissolved in IP buffer solution (100 mM NaCl, 1 mM EDTA, 50 mM TrIS-HCl, 0.5% NP-40, pH 8.0), and the supernatant was transferred to a pre-washed croton acylated resin (antibody resin No. Ptm-503 From Hangzhou Jingjing Biotechnology Co., LTD., PTM Bio), placed on a rotating shaker at 4 °C, gently shaken and incubated overnight. After incubation, the resin was washed 4 times with IP buffer solution and twice with deionized water. Finally, 0.1% trifluoroacetic acid eluent was used to elute the resin-bound peptide, which was eluted for three times. The eluent was collected and vacuum-dried. After being drained, the salt was removed according to C18 ZipTips instructions, and after being vacuum-frozen and drained, the liquid was supplied for mass analysis.ature away from light.

**LC-MS/MS analysis**

The peptides were dissolved by liquid chromatographic mobile phase A and separated by NanoElute ultra-high performance liquid system. Mobile phase A was an aqueous solution containing 0.1% formic acid and 2% acetonitrile. Mobile phase B contains 0.1% formic acid and 100% acetonitrile solution. Liquid phase gradient setting: 0-70 min, 3%-22% B; 70-85 min, 22%-33% B; 85-87 min, 33%-80% B; 87-90 min, 80%B, the flow rate was maintained at 300nL/min. The peptides were separated by ultra-performance liquid phase system and then injected into Capillary ion source for ionization and analyzed by timsTOF Pro mass spectrometry. The ion source voltage was set at 1.5kV, and the peptide parent ions and their secondary fragments were detected and analyzed using high-resolution TOF. The scanning range of secondary mass spectrometry was set to 100-1700. The data acquisition mode is parallel cumulative serial fragmentation (PASEF) mode. After a first-order mass spectrometry collection, the secondary spectra with the charge number of parent ions in the range of 0-5 were collected in PASEF mode for 10 times. The dynamic exclusion time of tandem mass spectrometry scanning was set to 30 s seconds to avoid repeated scanning of parent ions. The resulting MS/MS data were processed by using MaxQuant with integrated Andromeda search engine (version 1.5). False discovery rate thresholds for protein, peptide and modification sites were specified at 1%. Cutoffs for significant fold changes between cells by histone crotonylation were set as quantification ratios above 1.5 or below 0.67.

**Detection of FA concentration**

Mouse FA ELISA Kit (CUSABIO) was used to detect the level of FA in maternal serum and NTDs brain tissue. The maternal serum had been obtain by 3000rpm, 15min, 4℃ in advance. According to the manufacturer′s protocol, the levels of FA in control maternal serum were compared with those serum which from pregnant mice with NTDs.

**Human samples**

Clinical samples used in this study included 6 pairs of human NTDs and matched normal tissues were collected from Lvliang area in Shanxi Province with informed consent accepted by patients and their families. The International Classification of Diseases (ICD-10) was used to classify NTDs. Participants were diagnosed by local clinicians. All participates gave written informed consent. The details of surgical procedures are described in other studies (Zhang et al. 2018). The epidemiological studies are described in detail in our publication ( Pei et al. 2019; Xie et al. 2017). The Ethics Board of Capital Institute of Pediatrics approved the study protocol.

**Supp Figure legend**

Figure S1

(A, B) Folate content in (A) maternal serum or (B) fetal brain tissue of normal and MTX-induced mouse NTDs was harvested at E13.5, and analyzed by Elisa. Data are mean ± S.D. (n = 3). **P* < 0.05, ***P* < 0.01 by Student’s t test.

(C) left: Additional information of the human normal and NTD samples; right: Folate content in brain tissue of normal fetus and fetus with NTDs.

(D) Detection of ACSS2 in brain tissues from normal fetus and low-folate NTDs with low folate maternal serum. GAPDH was used as a loading control.

(E) Quantification of the ACSS2 signal intensity shown in scatter plot. Normal: n = 6; NTDs: n = 6; **p* < 0.05, by Student’s t test. NTDs vs. normal brains.

Figure S2

Peptide length distribution. Most of the peptides are distributed in 7-20 amino acids, which conforms to the general rule based on enzymatic hydrolysis and mass spectrometry fragmentation mode. The distribution of peptide length identified by mass spectrometry met the requirements of quality control.

Figure S3

(A) NE-4C were cultured in normal folate versus folate-free, (B) and supplementary folinic acid (50 mg/L) for 24 h and analyzed by western blot indicated antibody.

Figure S4

(A) SDS-PAGE analysis of lysates of mESC or (B) NE-4C in normal-folate and folate-free. Western blot analysis of cell lysate with anti-crotonyllysine antibodies.
